# Myocardial Infarction-Associated Extracellular Vesicle-Delivered miR-208b Affects the Growth of Human Umbilical Vein Endothelial Cells via Regulating CDKN1A

**DOI:** 10.1155/2021/9965639

**Published:** 2021-06-05

**Authors:** Wenyan Jiang, Qiaofeng Song, Zhihui Lu, Shuo Wang, Tao Liu, Xizhu Wang, Mei Wang

**Affiliations:** ^1^Department of Cardiology, Hebei Medical University, Shijiazhuang 050000, China; ^2^Department of Cardiology, Tangshan People's Hospital, Tangshan 063000, China; ^3^Department of Anesthesiology, Tangshan Workers' Hospital, Tangshan 063000, China; ^4^Department of Cardiology, The Second Hospital of Hebei Medical University, Shijiazhuang 050000, China

## Abstract

This study was aimed at investigating the effects of myocardial infarction- (MI-) associated extracellular vesicle- (EV-) delivered miR-208b on human umbilical vein endothelial cells (HUVECs). EVs were isolated and subsequently stained with PHK67. A dual-luciferase reporter gene assay was used to determine the target of miR-208b. Afterwards, HUVECs were transfected with either MI-associated EVs or miR-208b mimics, and cell viability, migration, and apoptosis were subsequently measured. Real-time quantitative polymerase chain reaction (RT-qPCR) was applied to determine the expressions of the tested genes. NanoSight, transmission electron microscopy, and western blotting showed that EVs were successfully isolated. Among the potential microRNA biomarkers for MI, miR-208b was chosen for subsequent experiments. We found that MI-associated EVs could be taken up by HUVECs and confirmed that *CDKN1A* was a direct target of miR-208b. Additionally, miR-208b mimics and MI-associated EVs significantly inhibited the viability and migration of HUVECs (*P* < 0.05) and promoted cell apoptosis, as well as reduced S phase and increased G2/M phase cell distribution. RT-qPCR revealed that both miR-208b mimics and MI-associated EVs upregulated the expressions of *CDKN1A*, *FAK*, *Raf-1*, *MAPK1*, and *Bax* but downregulated the expression of *Bcl2* and reduced the *Bcl2*/*Bax* ratio. Our study concludes that MI-associated EVs delivered miR-208b to HUVECs, and EV-delivered miR-208b could affect the growth of HUVECs by regulating the miR-208b/CDKN1A pathway; thus, miR-208b can be therefore served as important therapeutic targets for MI treatment.

## 1. Introduction

Myocardial infarction (MI), also known as a heart attack, is caused by a decrease or cessation of blood flow to the heart, resulting in damage of the cardiac muscles due to a lack of oxygen supply [1]. The clinical symptoms of MI include heart failure, shortness of breath, arrhythmias, shock, nausea, vomiting, and chest pain [2]. Formation of plaques in the intima of arteries is one of the primary causes of MI, as the rupture of plaques can lead to myocardial ischemia and subsequent infarction [3]. With the expansion of the ageing population in China, about 1 million deaths are caused by MI annually [4]. Currently, reperfusion therapy combined with guideline-directed medical treatment is used to treat MI and improve the survival of patients [3]. However, when cardiac shock occurs, the mortality rate is at least 40% [3, 5]. There is, therefore, an urgent need to further explore the pathogenesis of MI and identify new therapeutic targets for its treatment.

Extracellular vesicles (EVs) are released by various subtypes of cells and called exosomes, microvesicles, particles, intracellular bodies, apoptotic bodies, and many other names [6]. As carriers of proteins, lipids, and nucleic acids including DNA, RNA, and mRNA, they serve as communication tools among cells [7]. Previous studies have shown the usefulness of cancer cell-derived EVs in the diagnosis of many malignant tumors, such as breast cancer [8], pancreatic cancer [9], and glioblastoma [10]. Additionally, EVs also play important roles in cardiovascular disease (CVD). EVs are actually released from all cells in the cardiovascular system, and it was shown that stress conditions such as hypoxia or inflammation regulated their cargos and release with the target cells, thereby helping to improve or to impair cardiac function [11]. van Balkom et al. [12] showed that hsa-miR-214 can be delivered by endothelial cells exosomes, and transfer of this microRNA (miRNA) between endothelial cells could suppress cell senescence and promote angiogenesis. Another study indicated that miR-146a enriched in endothelial cells EVs that are efficiently taken up by cardiomyocytes had an essential role in the development of peripartum cardiomyopathy [13]. EVs isolated from stem cells of different origin have been reported to improve cardiac function after MI by reducing infarct size and fibrosis [14, 15]. EVs produced by cardiac cells or other cells can not only be internalized by neighboring cells, and thus play roles in CVD [16], but also can be released into the body fluids and can be considered as biomarkers of CVD.

MiRNAs are the most studied elements contained in EVs, and they are short (18–25 bp), noncoding RNAs that regulate protein synthesis by binding to mRNAs [17]. In the cardiovascular system, miRNAs are involved in the functional regulation of various cells, including myocardial cells, inflammatory cells, endothelial cells, smooth muscle cells, and fibroblasts, and have been proven to play an important role in CVDs [18]. A study by Agiannitopoulos et al. [19] showed that plasma miR-208b and miR-499 were significantly elevated in MI patients, proposing that they may serve as potential biomarkers for MI. Another study further suggested circulating miR-134 and miR-122-5p as potential diagnostic biomarkers for MI [20]. However, it is not clear whether MI-associated EVs, through the delivery of these miRNAs, have an impact on the development of CVD.

Since miR-499 released during MI has been investigated [21], this study determined the expression levels of miR-208b, miR-122-5p, and miR-134 in the EVs isolated from the plasma of MI patients and healthy individuals. The results showed that miR-208b level in the MI-associated EVs was significantly higher than that in the normal exosomes, which indicated that miR-208b enrichment may be closely associated with the development of MI. Therefore, miR-208b was selected for the following study. Additionally, endothelial dysfunction, such as dysregulated endothelial cell proliferation, migration, and apoptosis; endothelial barrier damage; and permeability changes, is an early physiological event in CVD [22]. Therefore, human umbilical vein endothelial cells (HUVECs) were utilized to explore the effects of MI-associated EV-delivered miR-208b on HUVECs alongside their mechanisms. These findings would help improve our understanding of CVD and provide novel therapeutic targets for the prevention and treatment of MI.

## 2. Materials and Methods

### 2.1. Isolation of EVs from Plasma

MI patients (*n* = 5) and healthy individuals (*n* = 3) were recruited from the Department of Cardiology of Tangshan People's Hospital. Inclusion criteria for the acute MI were shown as follows: (1) typical chest pain symptoms accompanied by dynamic changes in ST-segment of electrocardiogram, (2) elevated troponin or myocardial enzyme spectrum, and (3) coronary angiography showed occlusion of at least one important coronary artery. Additionally, patients with severe cardiac insufficiency, pulmonary heart disease, severe hepatic and renal insufficiency, severe infection, rheumatism and immune system diseases, blood system diseases, and malignant tumors and those taking immunosuppressants and antitumor drugs within 24 months should be excluded. The research protocol was approved by the Ethics Committee of Tangshan People's Hospital (approval number: rmyy-llks-2020-025), and informed consent was obtained from all subjects. The physiological and biochemical indexes of all subjects are shown in Table S1.

The plasma of all subjects was collected (5 mL), with that of MI patients taken at 4-6 hours after onset. The samples were centrifuged at 500 × g at 4°C for 10 min, followed by transfer of the supernatant to a new, sterile centrifuge tube for further centrifugation at 2000 × g at 4°C for 30 min. The supernatant was collected and was centrifuged again at 10000 × g at 4°C for 30 min. Following filtration with a 0.22 *μ*m sterile filter, the supernatant was centrifuged at ultrahigh speed at 120000 × g at 4°C for 1 h. Thereafter, the sediment was resuspended in 200 *μ*L sterile PBS, and the EVs were isolated and stored at -80°C.

### 2.2. Identification of Isolated EVs

The BCA protein assay kit (BOSTER Biological Technology Co., Ltd., CA, USA) was used to determine the concentration of isolated EVs, according to the manufacturer's instructions. Based on the method by Soares et al. [23], particle size distribution of EVs was measured using a NanoSight NS300 particle size analyzer (NTA, Malvern Panalytical, Malvern, UK). Thereafter, EVs were visualized by transmission electron microscopy (TEM, JEOL LTD, Peabody, MA, USA) as per a previous study [24]. Expressions of EV-specific proteins, including HSP70, CD9, and CD63, were assessed by western blotting as previously described [25]. Additionally, total RNAs from MI-associated and normal EVs were extracted using RNAiso Plus kit (Trizol, Takara Biomedical Technology Co., Ltd., Beijing, China) according to the manufacturer's protocol, and the levels of miR-208b, miR-122-5p, and miR-134 were determined using real-time quantification polymerase chain reaction (RT-qPCR). U6 was served as the housekeeping genes, and the levels of miR-208b, miR-122-5p, and miR-134 were calculated using 2^−*ΔΔ*Ct^ method [26]. The primer sequences of miR-208b, miR-122-5p, and miR-134 are displayed in Table 1.

### 2.3. Cell Culture and Transfection

HUVECs were purchased from Cell Bank, Chinese Academy of Sciences (Shanghai, China). The HUVECs were cultured in Dulbecco's modified Eagle's medium (DMEM, Thermo Fisher Scientific, Waltham, MA, USA) supplemented with 10% fetal bovine serum (FBS, Thermo Fisher Scientific), 100 kU/L penicillin (Thermo Fisher Scientific), and 100 mg/L streptomycin (Thermo Fisher Scientific) and then incubated in 5% carbon dioxide at 37°C.

Additionally, negative control (NC) mimics and miR-208b mimics were synthesized and provided by Yanzai Biotechnology Co., Ltd., Shanghai, China. To imitate cells with miR-208b enrichment, cell transfection with NC mimics or miR-208b mimics was performed, as previously described [27]. Briefly, the HUVECs were seeded into 6-well plates (5 × 10^5^ cells/well) and then transfected with 100 nM miR-208b mimics or 100 nM NC mimics using Lipofectamine 3000 (Thermo Fisher Scientific) based on the manufacturer's recommendations. After transfection for 6 h, the medium was replaced with complete medium. After culturing for another 48 h, total RNAs from the different cell groups were isolated, and the transfection efficiency was evaluated by determining the intake of miR-208b by RT-qPCR. The primer sequences of miR-208b are shown in Table 1.

### 2.4. Coculture of HUVECs and EVs

The EVs were labeled using the PKH67 staining kit (Sigma-Aldrich, USA) according to the manufacturer's instructions. Briefly, 60 *μ*g EVs were mixed with 970 *μ*L Diluent C and 4 *μ*L PKH67, incubated for 5 min, and mixed with 1 mL 1% bovine serum albumin (BSA, Sigma-Aldrich) to terminate dyeing. The mixture was then centrifuged at 120000 × g for 60 min, and the sediment was resuspended in 200 *μ*L PBS.

The HUVECs were seeded and cultured overnight in 24-well plates (5 × 10^4^ cells/well). The next day, the cells were rinsed 3 times with serum-free medium and then incubated in the same medium. Subsequently, 15 *μ*L PKH67-labeled EVs were added, and the cells were cultured in an incubator with 5% carbon dioxide at 37°C for 24 h and 48 h. After 3 PBS washes, the HUVECs were fixed with 4% paraformaldehyde (Beyotime Biotechnology, Shanghai, China), rewashed with PBS, and mounted in a DAPI-containing medium (Vector Laboratories, USA). The cells were eventually observed using a laser scanning confocal microscope (Leica Microsystems, Inc., USA).

### 2.5. Dual-Luciferase Reporter Gene Assay

The online analytic tool, TargetScan Human 7.1 (http://www.targetscan.org/vert_71/), was used to predict the target gene of miR-208b. The cyclin-dependent kinase inhibitor 1A (*CDKN1A*) 3′ untranslated region (3′UTR) sequence (Yanzai Biotechnology Co., Ltd., Shanghai, China) and the pGL3-basic vector (Yanzai Biotechnology Co., Ltd., Shanghai, China) were combined to establish the 3′UTR *CDKN1A* reporter plasmid (pGL3-CDKN1A). Briefly, 0.3 *μ*g pGL3-basic vector and 0.3 *μ*g pGL3-CDKN1A were cotransduced into 293T cells containing either 100 nM miR-208b mimics or 100 nM NC mimics by using Lipofectamine 3000 (Thermo Fisher Scientific) following the manufacturer's protocol. Luciferase reactivity was then assessed using a dual-luciferase reporter system (Promega, Madison, WI, USA).

### 2.6. Cell Viability and Migration Assays

The HUVECs were seeded into 96-well plates (1 × 10^4^ cells/well) and were divided into 4 groups: the control, miR-NC, miR-208b mimic, and MI-associated EV group. Cells of the miR-NC and miR-208b mimic groups were transfected with NC mimics and miR-208b mimics, respectively, while those of the control and MI-associated EV groups were treated with equal PBS and MI-associated EVs (50 *μ*g/mL, 100 *μ*g/mL, and 200 *μ*g/mL), respectively. After culturing for 24 h, 48 h, and 72 h, 10 *μ*L Cell Counting Kit-8 reagent (CCK-8, Beyotime Biotechnology) was added to each well, and the cells were incubated for 2 h after which the absorbance was measured at 450 nm using a microplate reader.

HUVEC migration of the different treatment groups was evaluated using Transwell chambers (pore size 8 *μ*m; Guangzhou Jet Bio-Filtration Co., Ltd., Guangzhou, China). Harvested cells were added into the upper Transwell chambers (1 × 10^4^ cells/well), while the lower Transwell chambers contained 500 *μ*L complete medium. After incubation for 24 h, the chambers were removed, and the cells were washed with PBS. The cells were then fixed with 500 *μ*L 4% paraformaldehyde for 20 min, washed with PBS 3 times, and stained with 0.5% crystal violet (Beyotime Biotechnology, Shanghai, China) for 20 min. After washing and air-drying, the images were observed under a microscope, and the relative number of cell migration was analyzed.

### 2.7. Cell Apoptosis and Cell Cycle Assays

Cells of the different treatment groups were evaluated for apoptosis using the Annexin V-FITC apoptosis assay kit (Beyotime Biotechnology) following the manufacturer's protocol. Briefly, the cells were centrifuged at 1000 × g for 5 min and were then resuspended in 195 *μ*L Annexin V-FITC binding solution. Subsequently, 5 *μ*L Annexin V-FITC and 10 *μ*L propidium iodide (PI) staining solutions were added. After incubation in the dark for 20 min, the cells were acquired by flow cytometry, and the apoptosis rate was calculated using the CellQuest software (Becton, Dickinson and Company, NJ, USA).

For the cell cycle assay, cells of each treatment group were centrifuged at 1000 × g for 5 min and were then resuspended in 200 *μ*L PBS. Afterwards, 4 mL 70% ethanol precooled to -20°C was added, and the cells were fixed in a 4°C refrigerator overnight. The fixed cells were then centrifuged at 1000 × g for 5 min and subsequently resuspended in PBS with 50 *μ*g/mL RNase A. After incubation at 37°C for 30 min, 20 *μ*L PI was added, and the mixture was incubated in the dark for another 30 min. Finally, a flow cytometer was utilized to observe cell cycle distribution.

### 2.8. RT-qPCR

The total RNA of each cell group was extracted using the RNAiso Plus kit (Trizol, Takara Biomedical Technology Co., Ltd., Beijing, China) according to the manufacturer's protocol. Thereafter, the extracted RNA was reverse transcribed into cDNA by using the PrimeScript™ II 1st Strand cDNA Synthesis kit (Takara, Dalian, China). RT-qPCR was initiated at 95°C for 2 min, followed by 40 cycles at 95°C for 15 s and at 60°C for 60 s. The sequences of all primers are shown in Table 1. Glyceraldehyde-3-phosphate dehydrogenase (*GAPDH*) was used as the housekeeping gene, and the relative mRNA levels of *CDKN1A*, Raf-1 proto-oncogene, serine/threonine kinase (*Raf-1*), focal adhesion kinase (*FAK*), BCL2 apoptosis regulator (*Bcl2*), BCL2-associated X, apoptosis regulator (*Bax*), and mitogen-activated protein kinase 1 (*MAPK1*) were calculated using the 2^−*ΔΔ*Ct^ method [26].

### 2.9. Statistical Analysis

Data are expressed as mean ± standard deviation (SD). One-way analysis of variance (ANOVA) followed by Bonferroni method was performed via GraphPad Prism 5 (GraphPad Software, San Diego, CA) to analyze the statistical significance between more than 2 groups, whereas the Student *t*-test was used for comparisons between 2 groups. *P* < 0.05 was considered statistically significant.

## 3. Results

### 3.1. Identification of EVs

**Figure 1:**
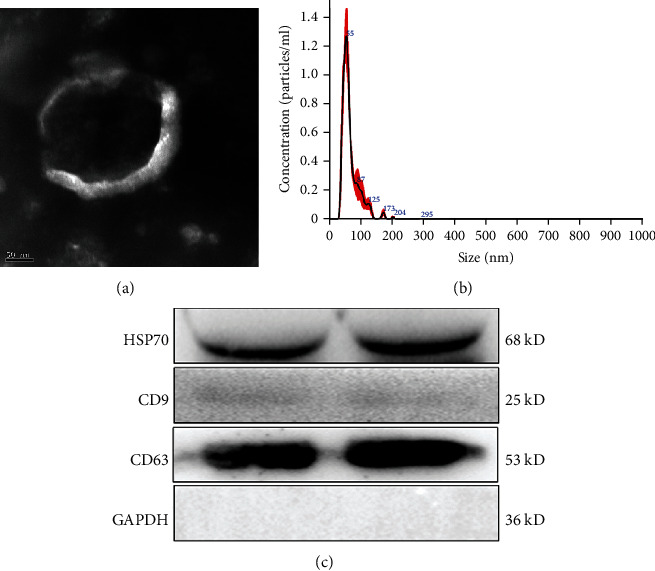
Identification of extracellular vesicles (EVs) isolated from the plasmas of myocardial infarction (MI). (a) The morphology of EVs observed by transmission electron microscopy. (b) The particle size distribution of EVs detected using the NanoSight NS300 particle size analyzer. (c) The surface markers of EVs (HSP70, CD9, CD63) were determined by western blotting, and the experiment was repeated twice (*n* = 2). Band 1 and band 2 are two duplicates.

EVs were isolated from the plasmas of MI patients and healthy individuals and were then identified by TEM, NTA, and western blotting. On TEM, the EVs displayed a nearly round- or cup-shaped morphology (Figure 1(a)). NTA analysis showed that the major peak of the isolated particles was about 55 nm, and the overall size distribution ranged from 50-200 nm (Figure 1(b)), which was in accordance with the size distribution of EVs previously reported [28]. In terms of western blotting, all EV-specific markers, HSP70, CD9, and CD63, were expressed (Figure 1(c)). These results altogether suggested that EVs were successfully isolated.

### 3.2. Screen of miRNAs and Cell Transfection Efficiency

**Figure 2:**
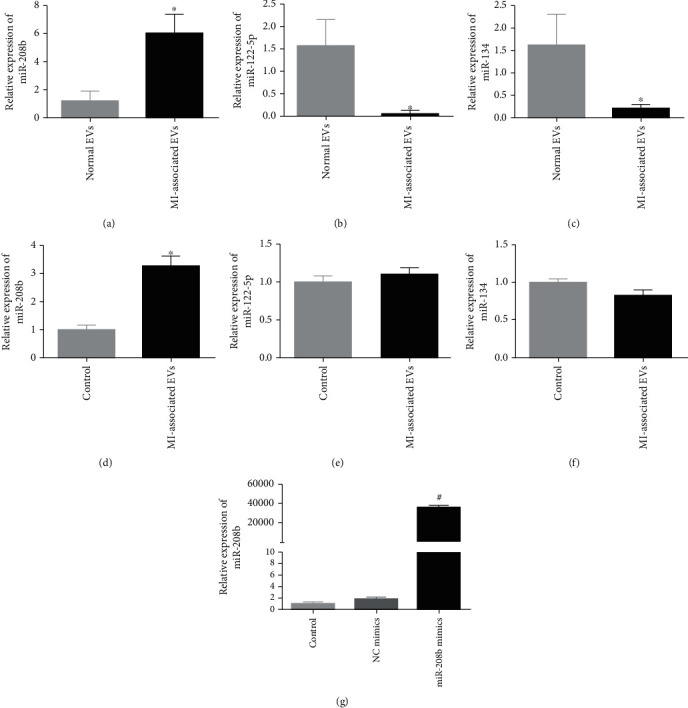
The levels of miR-208b (a), miR-122-5p (b), and miR-134 (c) in the EVs isolated from the plasmas of MI patients and healthy individuals, detected by real-time quantitative PCR (RT-qPCR). Normal EVs: EVs isolated from the plasma of the healthy individuals; MI-associated EVs: EVs isolated from the plasma of the MI patients. ^∗^*P* < 0.05, compared with normal EVs, *n* = 3. The levels of miR-208b (d), miR-122-5P (e), and miR-134 (f) in the control and MI-associated EV-transfected HUVECs. Control: HUVECs without treatment; MI-associated EVs: HUVECs transfected with MI-associated EVs. ^∗^*P* < 0.05, compared with the control group, *n* = 3. (g) The cell transfection efficiency evaluated based on the level of miR-208b. ^#^*P* < 0.05, compared with the control group, *n* = 3.

miR-208b, miR-122-5p, and miR-134 have been reported to be closely associated with MI; and their levels in the EVs isolated from MI patients and healthy individuals were hence assessed using RT-qPCR. miR-208b amount in the MI-associated EVs was significantly higher than that in the controls (*P* < 0.05, Figure 2(a)), while the amounts of miR-122-5p and miR-134 were significantly lower (*P* < 0.05, Figures 2(b) and 2(c)). Furthermore, levels of miR-208b, miR-122-5p, and miR-134 were further measured in both normal HUVECs and those transfected with MI-associated EVs. No significant differences in miR-122-5p and miR-134 levels were shown between the two groups (*P* > 0.05, Figures 2(e) and 2(f)), whereas miR-208b level in the EV-transfected group was significantly enriched compared with that in the normal HUVECs (*P* < 0.05, Figure 2(d)). These results indicated the ability of MI-associated EVs in delivering miR-208b to cells. Therefore, miR-208b was selected for the rest of our study.

In addition, miR-208b-enrichment cells were imitated by miR-208b mimic transfection, and the transfection efficiency was evaluated in terms of miR-208b content in the cells. No significant differences in miR-208b content were observed between the control and NC mimic groups (*P* > 0.05, Figure 2(g)). In contrast, a significant increase in miR-208b content was demonstrated in the miR-208b mimic group compared with the control group (36509.09 ± 2305.34 vs. 1.03 ± 0.31, respectively, *P* < 0.05, Figure 2(g)). These results indicated that HUVEC lines with enriched miR-208b were successfully established.

### 3.3. Cellular Uptake of EVs in HUVECs

**Figure 3:**
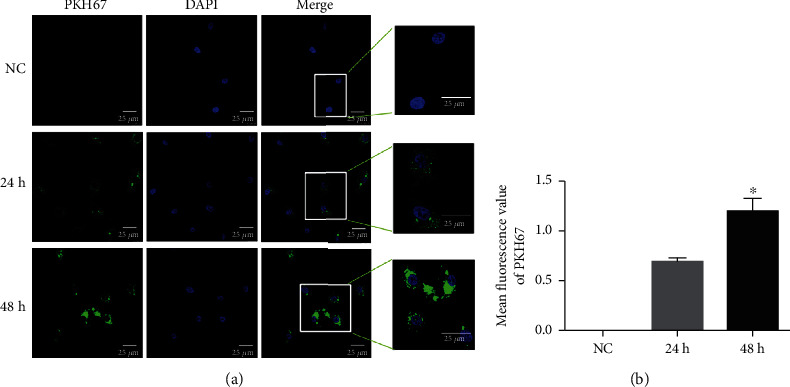
PKH67-labeled MI-associated EVs (green) taken up by HUVECs after coculture for 24 h and 48 h. (a) Immunofluorescence images were obtained using a laser scanning confocal microscope (at the magnification of 400x). (b) Mean fluorescence values of PKH67 analyzed by ImageJ software. ^∗^*P* < 0.05, compared with cultured for 24 h, *n* = 3.

MI-associated EVs were stained green by PKH67, and the nuclei of HUVECs were stained blue by DAPI. After coculture for 24 h and 48 h, it was found that most HUVECs exhibited intracellular green fluorescence (Figure 3(a)), and the fluorescence intensity after 48 h was significantly stronger than that after 24 h (*P* < 0.05, Figure 3(b)). This indicated that MI-associated EVs could be taken up by HUVECs and that cell uptake of EVs increased with culture time.

### 3.4. miR-208b Directly Binds to CDKN1A

**Figure 4:**
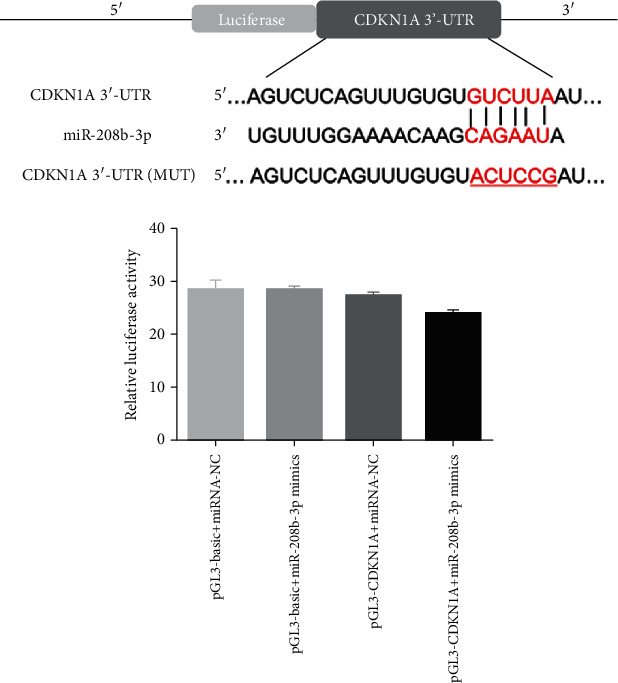
*CDKN1A* was the target gene of miR-208b. (a) TargetScan Human 7.1 was utilized to predict the target gene of miR-208b. (b) The relative luciferase activity was measured after 293T cells were cotransfected with either miR-208b-3p mimics or miRNA-NC and pGL3-CDKN1A luciferase vector or pGL3-basic vector, *n* = 3. ^∗^*P* < 0.05, compared with the pGL3 − CDKN1A + miRNA − NC group.

The online analysis tool TargetScan Human 7.1 predicted *CDKN1A* as a potential target gene of miR-208b, as several miR-208b binding sites were found in the *CDKN1A* 3′UTR (Figure 4(a)). A dual-luciferase reporter gene assay was performed to confirm this. The relative luciferase activity of pGL3-CDKN1A was significantly lower after transfection with miR-208b-3p mimics than with miRNA-NC (*P* < 0.05, Figure 4(b)). Additionally, no significant difference in relative luciferase activity was observed among pGL3-CDKN1A transfected with miRNA-NC, pGL3-basic transfected with miR-208b-3p mimics, and pGL3-basic transfected with miRNA-NC (*P* > 0.05, Figure 4(b)). These results confirmed *CDKN1A* as the target gene of miR-208b.

### 3.5. Analyses of Cell Viability and Migration

**Figure 5:**
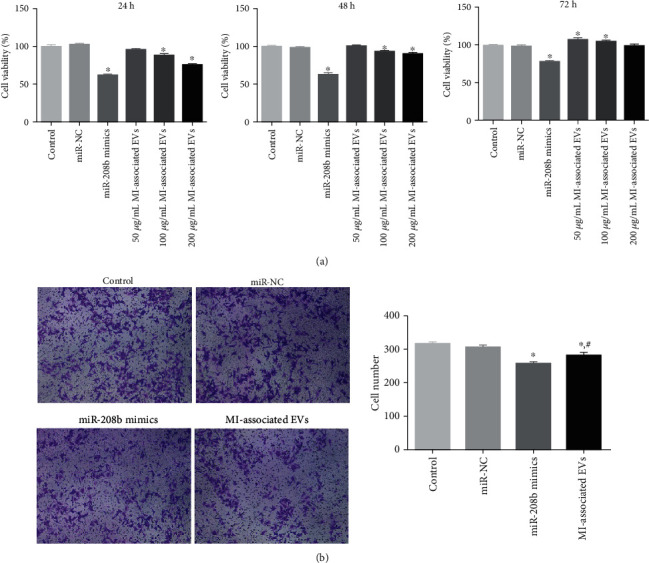
(a) The viability of HUVECs following the different treatments for 24 h, 48 h, and 72 h, determined by Cell Counting Kit-8 (CCK-8), *n* = 3. (b) HUVECs were treated with 100 *μ*g/mL MI-associated EVs for 24 h, and the migration of HUVECs following the different treatments was determined by the Transwell assay. Left: images showing crystal violet staining under a microscope (at the magnification of 100x). Right: cell number of the different groups, *n* = 3. ^∗^*P* < 0.05, compared with the control group. ^#^*P* < 0.05, compared with the miR-208b mimic group.

CCK-8 was used to determine the viability of HUVECs following the different treatments. No significant difference in cell viability was observed between the control and miR-NC groups (Figure 5(a)). After the 24 h culture, viability of the 50 *μ*g/mL MI-associated EV group was lower than that of the control group, but there was no significant difference (*P* > 0.05, Figure 5(a)). After the 24 h and 48 h culture, viabilities of the miR-208b mimics and the 100 *μ*g/mL and 200 *μ*g/mL MI-associated EV groups were significantly reduced compared to those of controls (*P* < 0.05, Figure 5(a)). However, after the 72 h culture, viabilities of the 50 *μ*g/mL and 100 *μ*g/mL MI-associated EVs groups were significantly higher than those of the control group. Thus, HUVEC treatment with 100 *μ*g/mL MI-associated EVs at a duration of 24 h was chosen for subsequent experiments.

Thereafter, the effects of miR-208b and MI-associated EVs on cell migration were evaluated using the Transwell assay. Mean cell numbers of the control and miR-NC groups were 316 ± 4.5 and 306 ± 6.4, respectively, which showed no significant difference (*P* > 0.05, Figure 5(b)). After transfection with miR-208b mimics and MI-associated EVs, the number reduced to 256 ± 9.7 and 281 ± 12, respectively, with that of the miR-208b mimic group being significantly higher than that of the MI-associated EV group (*P* < 0.05, Figure 5(b)). Results of both the CCK-8 and Transwell assays showed that miR-208b and MI-associated EVs inhibited both HUVEC viability and migration.

### 3.6. Analyses of Cell Apoptosis and Cycle

**Figure 6:**
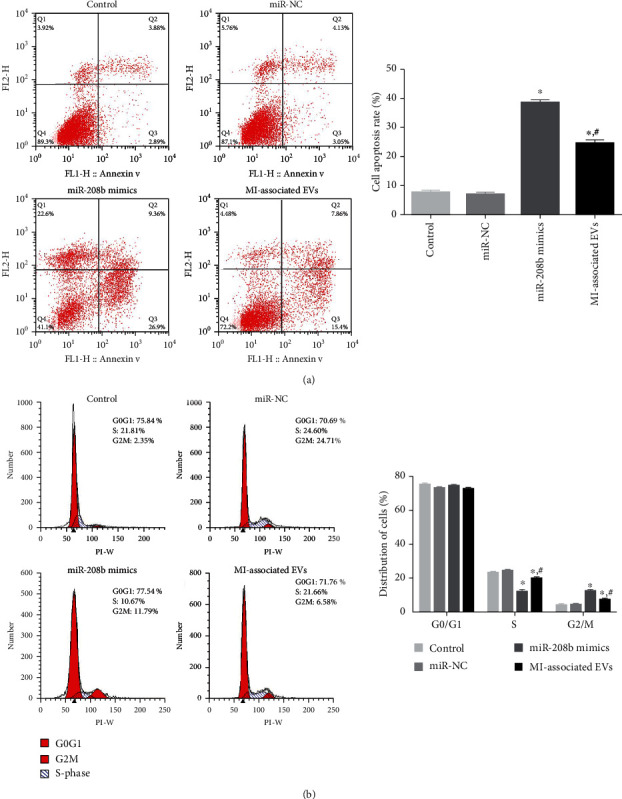
(a) The apoptosis rate of HUVECs in the different groups, determined by flow cytometry. Left: representative images acquired by flow cytometry. Right: apoptosis rates of the different groups, *n* = 3. (b) The cycle progression of HUVECs following the different treatments, examined using flow cytometry. Left: images obtained by flow cytometry. Right: distribution of cells of the different groups in the different phases, *n* = 3. ^∗^*P* < 0.05, compared with the control group. ^#^*P* < 0.05, compared with the miR-208b mimic group.

The effects of miR-208b and MI-associated EVs on cell apoptosis and cycle progression were investigated by flow cytometry. Compared with the control group, apoptosis rates of both the miR-208b mimic and MI-associated EV groups were significantly higher (*P* < 0.05, Figure 6(a)), with those of the miR-208b mimic group higher than those of the MI-associated EV group. Results of the cell cycle analysis showed no significant difference in cell distribution of the G0/G1 phase among the groups (Figure 6(b)). After transfection with miR-208b mimics and MI-associated EVs, the number of cells in the S phase was lower compared to controls, but it was higher in the G2/M phase (Figure 6(b)). Besides, the change trend after miR-208b mimic transfection was more significant compared with that after MI-associated EV treatment (*P* < 0.05, Figure 6(b)). The results indicated that miR-208b and MI-associated EVs promoted cell apoptosis, as well as decreased S phase and increased G2/M phase cell distribution.

### 3.7. RT-qPCR Analysis

**Figure 7:**
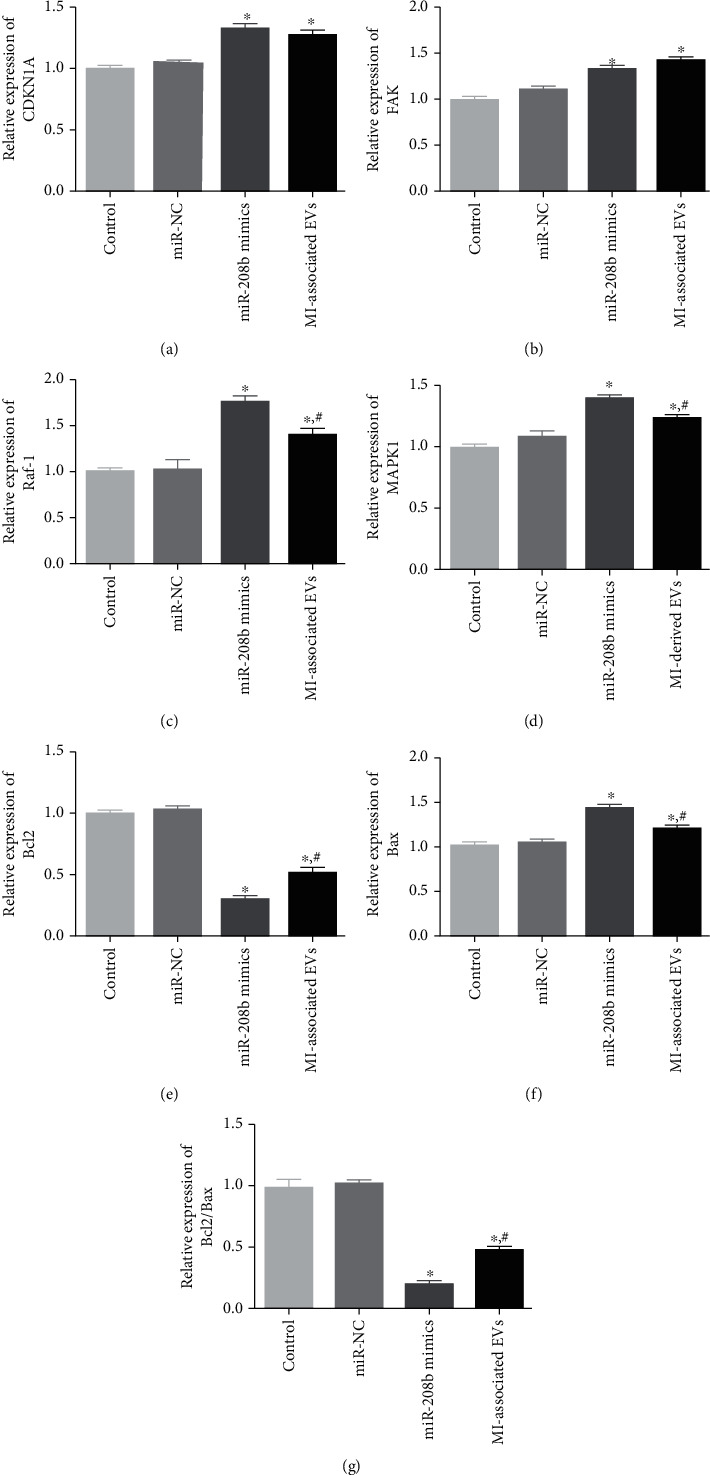
The expression levels of *CDKN1A* (a), *FAK* (b), *Raf-1*(c), *MAPK1* (d), *Bcl2* (e), *Bax* (f), and *Bcl2*/*Bax* ratio (g) of the different groups, measured by RT-qPCR, *n* = 3. ^∗^*P* < 0.05, compared with the control group. ^#^*P* < 0.05, compared with the miR-208b mimic group.

In order to understand the molecular mechanisms to which miR-208b and MI-associated EVs impact HUVEC growth, RT-qPCR was employed to determine the relative expression levels of *CDKN1A*, *Raf-1*, *FAK*, *Bcl2*, *Bax*, and *MAPK1*. No significant differences were demonstrated in the expression levels of all tested genes between the control and miR-NC groups. In both the miR-208b mimic and MI-associated EV groups, expression levels of *CDKN1A* and *FAK* were significantly upregulated compared with the control group (*P* < 0.05, Figures 7(a) and 7(b)). Expressions of *Raf-1* and *MAPK1* were also significantly upregulated (*P* < 0.05), with significantly higher expression levels in the miR-208b mimic group than in the MI-associated EV group (*P* < 0.05, Figures 7(c) and 7(d)). In terms of *Bcl2*, expression levels were significantly downregulated in the miR-208b mimic and MI-associated EV groups (*P* < 0.05, Figure 7(e)); however, an opposite trend was shown for *Bax* expression (Figure 7(f)). The *Bcl2*/*Bax* ratio was subsequently calculated, and significantly lower values were observed in both the miR-208b mimic and MI-associated EV groups, compared to the control group (*P* < 0.05, Figure 7(g)).

## 4. Discussion

MI caused by myocardial ischemia is a major cause of morbidity and mortality worldwide, seriously affecting people's health and quality of life [29]. EVs delivered miRNAs, which act as endocrine, autocrine, and paracrine factors, have been reported to participate in the progression of diseases by mediating intercellular communication [30]. Our study is the first to explore the effects of MI-associated EVs delivered miRNAs on HUVECs. By comparing the levels of miR-208b, miR-122-5p, and miR-134 between MI-associated and normal EVs, miR-208b was found to be enriched in the MI-associated EVs and was hence selected for further experiments. PKH67 staining subsequently demonstrated that EVs could be taken up by HUVECs, and the dual-luciferase reporter gene assay further showed that *CDKN1A* was the target gene of miR-208b. The HUVECs were then transfected with either MI-associated exosomes or miR-208b mimics, which demonstrated that both miR-208b mimics and MI-associated EVs inhibited HUVEC viability and migration, promoted cell apoptosis, and led to decreased S phase and increased G2/M phase cell distributions. Finally, RT-qPCR revealed that both miR-208b mimics and MI-associated EVs upregulated *CDKN1A*, *FAK*, *Raf-1*, *MAPK1*, and *Bax* but downregulated *Bcl2* and reduced the *Bcl2*/*Bax* ratio. Our results suggested that MI-associated EV-delivered miR-208b may affect the growth of HUVECs by regulating apoptosis-related genes and *CDKN1A*, thereby influencing the progression of MI.

Previous studies have indicated that EVs serve as a natural carrier system that transport mRNA, miRNA, and proteins among cells and are involved in cardiovascular cell-to-cell communications [31, 32]. A study of Leroyer et al. [33] showed that endothelial cells release EVs enriched with miR-143/145, which can be taken up by smooth muscle cells, and regulate gene expressions in the recipient cells, thus playing a role in atherosclerosis. Another study has shown that cardiac fibroblasts secrete EVs enriched in miR-21∗, which can be shuttle to cardiomyocytes, and act as a key paracrine signaling mediator for cardiac hypertrophy [34]. Therefore, EVs can deliver miRNAs to other cells to perform their functions. MicroRNAs play key roles in MI by regulating apoptosis, necrosis, and autophagy. Li et al. [35] reported that miR-208b was highly expressed in MI patients and that p21 was the direct target of miR-208b. In our study, miR-208b was also significantly enriched in MI patients than that in the healthy individuals, and miR-208b mimics exerted similar effects as MI-associated EVs. Both miR-208b mimics and MI-associated EVs suppressed HUVEC viability and migration and induced cell apoptosis. Additionally, they influenced the cell cycle by decreasing the distribution of the S phase, while increasing that of the G2/M phase. A study by Zhou et al. [36] showed that miR-208b could alleviate heart injuries in MI rat models and inhibit postinfarction myocardial fibrosis, which implied that miR-208b may play an important role in MI. Another study demonstrated that exosomes isolated from adipose-derived stem cells with miR-126 enrichment prevented myocardial damage by protecting myocardial cells against apoptosis, inflammation, and fibrosis, as well as by promoting angiogenesis [37]. These findings, combined with our results, support the speculation that EVs can deliver miR-208b to HUVECs and that EV-delivered miR-208b may influence the development of MI by regulating cell viability, migration, apoptosis, and cell cycle events.

To further understand the molecular mechanisms to which MI-associated EVs and miR-208b affect HUVEC growth, the target gene of miR-208b was predicted, and the expressions of *CDKN1A*, *FAK*, *Raf-1*, *MAPK1*, *Bcl2*, and *Bax* were measured using RT-qPCR. By employing the online tool, TargetScan Human 7.1, and the dual-luciferase reporter gene assay, *CDKN1A* was found to be the target gene of miR-208b; furthermore, RT-qPCR revealed that miR-208b mimics and MI-associated EVs significantly upregulated *CDKN1A* expression. *CDKN1A* encodes for p21 protein and belongs to the Cip/Kip family [38]. *CDKN1A* is not only a tumor suppressor, a cell cycle inhibitor, and a senescence inducer but also a key regulator of cell migration, apoptosis, differentiation, DNA repair, and transcription [39, 40]. In addition, *CDKN1A* is vital for G2/M transition and mitotic progression [41]. Our results showed that *CDKN1A* upregulation promoted cell apoptosis by decreasing the S phase and increasing G2/M phase cell distributions. Gang et al. [42] indicated that *CDKN1A* overexpression suppressed the proliferation and invasion of human fibroblast-like synoviocytes and arrested the cells in the G0/G1 phase. Another study has shown that *CDKN1A* overexpression increased the proportion of the G0/G1 phase cells, decreased that of the S phase cells, and elevated cell apoptosis in ovarian cancer cells [43]. These results suggested that EV-delivered miR-208b could affect cell apoptosis and cycle progression by mediating the miR-208b/CDKN1A pathway.

In addition, our study showed that transfection with either miR-208b mimics or MI-associated EVs significantly upregulated the expressions of *FAK*, *Raf-1*, *MAPK1*, and *Bax*, while significantly downregulating those of *Bcl2* and significantly reducing the *Bcl2*/*Bax* ratio. *FAK*, an integrin-associated protein tyrosine kinase, plays a major role in cellular communication, especially in cellular signaling systems [44]. Previous studies have reported that *FAK* is overexpressed in many cancer cells and is closely associated with cell survival, proliferation, migration, and invasion [45, 46]. Crowe and Ohannessian further demonstrated that *FAK* regulated migration and invasion of squamous cell carcinoma lines by activating MPKAs [47]. *MAPK1* is a key member of the MAPK/ERK pathway and participates in multifarious pathological processes of cells [48, 49], while *Raf-1* is a serine/threonine protein kinase that plays an important role in cell proliferation [50]. The Raf-1/MAPK1,3 pathway has been reported to be involved in the control of Bcl2 family proteins and prevent the activation of Caspase 9 in the apoptosome complex [51]. *Bcl2* is an antiapoptotic gene, while *Bax* is a proapoptotic gene. Their ratio determines the trend of cell apoptosis, with u-regulation of the *Bcl-2/Bax* ratio reflecting inhibited apoptosis and vice versa [52]. Findings of these studies, alongside ours, support the speculation that EV-delivered miR-208b might regulate apoptosis-related genes (*Bcl2* and *Bax*) via the FAK/MAPK1/Raf-1 pathway, thereby influencing HUVEC growth.

However, this study had several limitations. The number of clinical samples is small; therefore, our findings on the effects of miR-208b on CVD warrant further validation in larger samples and animal models and *in vivo* evaluation. The protein expressions of CDKN1A, Raf-1, FAK, Bcl2, MAPK1, p-MAPK1, and p-FAK should be analyzed using western blot, and the relationship of miR-208b with *CDKN1A*, *Raf-1*, *FAK*, *Bcl2*, *Bax*, *and MAPK1* also require further verification by a series of rescue experiments. Additionally, the cell source of EVs extracted from the plasmas of MI patients and the factors contributing to the miR-208b enrichment in patients with acute MI need to be further explored.

## 5. Conclusions

In conclusion, MI-associated EVs can deliver miR-208b to HUVECs, and the enrichment of miR-208b may suppress cell viability and migration, as well as promote cell apoptosis by regulating apoptosis-related genes (*Bcl2* and *Bax*) and the FAK/MAPK1/Raf-1 pathway. Additionally, EV-delivered miR-208b can induce cell apoptosis and cycle arrest in HUVECs by mediating the miR-208b/CDKN1A pathway. Our findings imply that EV-delivered miR-208b represents a promising target for the improvement of MI treatment, and targeting the miR-208b/CDKN1A pathway may function as a novel therapeutic approach in CVD.

## Figures and Tables

**Table 1:** The sequences of all primers.

Primer	Sequence (5′-3′)
hsa-miR-208b-3p-RT	GTCGTATCCAGTGCAGGGTCCGAGGTATTCGCACTGGATACGACACAAAC
hsa-miR-208b-3p-F	GCGGCATAAGACGAACAAAAG
hsa-miR-122-5p-RT	GTCGTATCCAGTGCAGGGTCCGAGGTATTCGCACTGGATACGACCAAACA
hsa-miR-122-5p-F	GGCTGGAGTGTGACAATGG
hsa-miR-134-5p-RT	GTCGTATCCAGTGCAGGGTCCGAGGTATTCGCACTGGATACGACCCCCTC
hsa-miR-134-5p-F	GCTGTGACTGGTTGACCA
U6-F	CTCGCTTCGGCAGCACA
U6-R	AACGCTTCACGAATTTGCGT
CDKN1A-hF	TGTCCGTCAGAACCCATGC
CDKN1A-hR	AAAGTCGAAGTTCCATCGCTC
Raf-1-hF	GGGAGCTTGGAAGACGATCAG
Raf-1-hR	ACACGGATAGTGTTGCTTGTC
FAK-hF	GCTTACCTTGACCCCAACTTG
FAK-hR	ACGTTCCATACCAGTACCCAG
Bcl-2-hF	AGTACCTGAACCGGCACCT
Bcl-2-hR	CCACCAGGGCCAAACTGAGCA
Bax-hF	CATATAACCCCGTCAACGCAG
Bax -hR	GCAGCCGCCACAAACATAC
MAPK1-hF	TCTGGAGCAGTATTACGACCC
MAPK1-hR	CTGGCTGGAATCTAGCAGTCT
GAPDH-hF	TGACAACTTTGGTATCGTGGAAGG
GAPDH-hR	AGGCAGGGATGATGTTCTGGAGAG

## Data Availability

The dataset used and/or analyzed during the current study are available from the corresponding author on reasonable request.

## Supplementary Materials

Supplementary Materials Supplementary Table S1: the physiological and biochemical indexes of myocardial infarction (MI) patients and healthy individuals.
