# Supplementary material for: Myocardial Infarction-Associated Extracellular Vesicle-Delivered miR-208b Affects the Growth of Human Umbilical Vein Endothelial Cells via Regulating CDKN1A
Source: Biomed Res Int. 2021 Jun 5;2021:9965639. doi: 10.1155/2021/9965639 (PMC8203352; doi:10.1155/2021/9965639)
Supplement: Supplementary Materials — Supplementary Table S1: the physiological and biochemical indexes of myocardial infarction (MI) patients and healthy individuals. [file 9965639.f1.docx]

**Table S1** The physiological and biochemical indexes of myocardial infarction (MI) patients and healthy individuals

| Types | Number | Sex | Age | blood pressure | BMI | Course | hs-c  TNI(ng/mL) | TG(mmol/L) | TC(mmol/L) | HDL (mmol/L) | LDL(mmol/L) | ALT(IU/L) | GGT(IU/L) | CK (IU/L) | Scr (μmol/L) | CKMB (IU/L) | Spherocyte (×10^3^/cm^3^) | Leukocyte | Ca^2+^(mg/dL) | Cl(mg/dL) | Na(mmol/L) | K(mmol/L) |
| --- | --- | --- | --- | --- | --- | --- | --- | --- | --- | --- | --- | --- | --- | --- | --- | --- | --- | --- | --- | --- | --- | --- |
| MI | 1 | Male | 54 | 120/85 | 24.2 | 24 h | 10.72 | 0.85 | 3.59 | 0.87 | 2.46 | 24 | 76 | 742 | 54 | 89 | 237 | 9.35 | 2.16 | 106.4 | 142.1 | 3.69 |
|  | 2 | Male | 69 | 102/54 | 21.9 | 4.5 h | 23.28 | 1.43 | 4.57 | 1.06 | 3.15 | 54 | 137 | 1390 | 54 | 143 | 241 | 8.31 | 2.08 | 107.2 | 137.2 | 3.89 |
|  | 3 | Female | 64 | 161/91 | 19.8 | 15 h | 6.26 | 1.39 | 4.19 | 1.2 | 2.68 | 24 | 40 | 251 | 57 | 29 | 213 | 6.33 | 2.24 | 107.6 | 140.7 | 4 |
|  | 4 | Female | 59 | 113/64 | 23.1 | 5 h | 6.14 | 1.8 | 4.82 | 0.94 | 3.26 | 61 | 185 | 243 | 50 | 43 | 266 | 10.13 | 2.24 | 109.9 | 142.5 | 3.98 |
|  | 5 | Male | 53 | 189/109 | 22.1 | 13 h | 8.68 | 1.17 | 4.99 | 1.13 | 3.45 | 22 | 45 | 507 | 55 | 61 | 219 | 7.53 | 2.35 | 106.8 | 140.8 | 4.01 |
| healthy | 1 | Male | 53 | 130/70 | 22.6 | / | 0.015 | 1.10 | 3.5 | 1.81 | 3.48 | 20 | 17 | 103 | 55 | 15 | 224 | 5.6 | 2.24 | 104 | 140 | 3.56 |
|  | 2 | Female | 33 | 125/70 | 21.1 | / | 0.001 | 0.98 | 4.5 | 1.43 | 2.95 | 22 | 18 | 77 | 57 | 13 | 210 | 7.8 | 2.42 | 103 | 142 | 4.41 |
|  | 3 | Female | 33 | 130/75 | 28.04 | / | 0.001 | 1.51 | 4.6 | 1.90 | 3.53 | 25 | 22 | 97 | 59 | 13 | 278 | 7.4 | 2.26 | 106 | 141 | 4.16 |

BMI: Body Mass Index; TG: triglyceride; TC: total cholesterol; HDL: high density lipoprotein; LDL: low density lipoprotein; ALT: glutamic-pyruvic transaminase; GGT: gamma-glutamyl transpeptidase; CK: creatine kinase; Scr: serum creatinine; CKMB: creatine kinase MB; Ca^2+^: calcium ion; Cl: chlorine; Na: sodium; K: potassium.
